# Neurodegenerative Disorder Risk in Idiopathic REM Sleep Behavior Disorder: Study in 174 Patients

**DOI:** 10.1371/journal.pone.0089741

**Published:** 2014-02-26

**Authors:** Alex Iranzo, Ana Fernández-Arcos, Eduard Tolosa, Mónica Serradell, José Luis Molinuevo, Francesc Valldeoriola, Ellen Gelpi, Isabel Vilaseca, Raquel Sánchez-Valle, Albert Lladó, Carles Gaig, Joan Santamaría

**Affiliations:** 1 Neurology Service, Hospital Clínic de Barcelona, IDIBAPS, Barcelona, Spain; 2 CIBERNED, Barcelona, Spain; 3 Neurological Tissue Bank, Biobanc-Hospital Clinic, IDIBAPS, Barcelona, Spain; 4 Otorhinolaryngology Service, Hospital Clínic de Barcelona, CIBER Enfermedades Respiratorias, Bunyola, Spain; Oslo University Hospital, Norway

## Abstract

**Objective:**

To estimate the risk for developing a defined neurodegenerative syndrome in a large cohort of idiopathic REM sleep behavior disorder (IRBD) patients with long follow-up.

**Methods:**

Using the Kaplan-Meier method, we estimated the disease-free survival rate from defined neurodegenerative syndromes in all the consecutive IRBD patients diagnosed and followed-up in our tertiary referal sleep center between November 1991 and July 2013.

**Results:**

The cohort comprises 174 patients with a median age at diagnosis of IRBD of 69 years and a median follow-up of four years. The risk of a defined neurodegenerative syndrome from the time of IRBD diagnosis was 33.1% at five years, 75.7% at ten years, and 90.9% at 14 years. The median conversion time was 7.5 years. Emerging diagnoses (37.4%) were dementia with Lewy bodies (DLB) in 29 subjects, Parkinson disease (PD) in 22, multiple system atrophy (MSA) in two, and mild cognitive impairment (MCI) in 12. In six cases, in whom postmortem was performed, neuropathological examination disclosed neuronal loss and widespread Lewy-type pathology in the brain in each case.

**Conclusions:**

In a large IRBD cohort diagnosed in a tertiary referal sleep center, prolonged follow-up indicated that the majority of patients are eventually diagnosed with the synucleinopathies PD, DLB and less frequently MSA. IRBD represented the prodromal period of these conditions. Our findings in IRBD have important implications in clinical practice, in the investigation of the early pathological events occurring in the synucleinopathies, and for the design of interventions with potential disease-modifying agents.

## Introduction

REM sleep behavior disorder (RBD) is a parasomnia characterized by dream-enacting behaviors associated with REM sleep without muscle atonia [1], [2], [3]. Longitudinal studies conducted in sleep centers have shown that patients diagnosed with the idiopathic form of RBD (IRBD) may eventually be diagnosed with a neurological disorder such as Parkinson disease (PD) and dementia with Lewy bodies (DLB) [4], [5], [6], [7], [8]. In a recent study we assessed the first 44 IRBD individuals diagnosed in our tertiary referral sleep center between 1991 and 2003 and found that after a median follow-up of 10.5 years, 82% were diagnosed with PD, DLB and less frequently multiple system atrophy (MSA) and mild cognitive impairment (MCI). The estimated risk of defined neurodegenerative syndrome from the diagnosis of IRBD was 34.8% at five years, 73.4% at ten years and 92.5% at 14 years [8]. These findings, in a group of 44 individuals with long and close follow-up, indicate that most IRBD subjects develop a synucleinopathy with time. Since additional IRBD patients have been diagnosed and followed-up in our sleep center, we aimed to confirm our initial observation in a larger cohort of subjects that comprises all the 174 consecutive IRBD cases diagnosed up to July 2013. Confirmation of our previous findings in a much larger cohort would strongly support the notion that IRBD is a manifestation of prodromal PD, DLB or MSA. This would carry important implications in patient management, in understanding the pathophysiology of the various disorders associated with abnormal synuclein deposition, and could provide the opportunity to test disease-modifying strategies before the onset of motor and cognitive symptoms.

## Methods

In the present study we determined the risk of development a defined neurodegenerative syndrome in all the consecutive 174 subjects that were diagnosed with IRBD between November 1991 and July 2013.

### Patients’ Assessment

In all instances the diagnosis of IRBD required history of dream-enacting behaviors, video-polysomnography detection of REM sleep with increased muscular activity associated with abnormal behaviors, absence of known neurodegenerative diseases, lack of motor and cognitive complaints, normal neurological examination, and RBD not explained by a brain lesion (e.g., stroke, demyelinating plaque, encephalitis) or by the introduction or withdrawal of any medication or substance (e.g., antidepressants, beta-blockers) [1], [2], [3].

The methods of clinical assessment at first and follow-up visits have been described elsewhere [7], [8]. In brief, patients were referred to our sleep center where they or their bed partners reported abnormal sleep behaviors. When the diagnosis of IRBD was confirmed by video-polysomnography, patients underwent routine follow-up visits every 3–12 months in our sleep center where neurologists experienced in both sleep and neurodegenerative disorders assessed sleep quality. If during follow-up visits neurologists, patients or relatives noted the appearance of symptoms or signs suggestive of an emerging neurological disorder, the patient was referred for detailed assessment to our movement disorders or memory disorders units. Neurologists from these units then formally assessed patients through detailed clinical history, comprehensive neurological examination and neuropsychological tests when cognitive impairment was suspected. Diagnoses criteria applied were those accepted for PD, dementia, DLB, MSA and MCI [9], [10], [11], [12], [13]. If patients were not able or willing to attend our clinic, they were offered to undergo home and home-nursing visits by a neurologist (A.I) and a neuropsychologist (M.S) from our sleep center.

In the current study, to assess the presence and nature of a defined neurodegenerative syndrome, clinical records were reviewed, and all available patients were scheduled at our clinic (or at their homes or nursing homes) between February and August 2013. During these assessments, we conducted a detailed medical history, a neurological examination and neuropsychological testing when cognitive symptoms were first reported or noticed. The clinical outcome of the patients (those who remained with IRBD or those who were eventually diagnosed with a defined neurodegenerative syndrome) was determined in all cases by means of direct, face-to-face clinical interviews, neurological examinations and neuropsychological tests. Diagnoses were not done by telephone interviews. We also reviewed the neuropathological findings (according to a protocol previously described [8]) of six patients from our cohort who died and donated their brain to the Neurological Tissue Bank of the Biobank-Hospital Clinic de Barcelona-IDIBAPS.

The study was approved by the Hospital Clinic of Barcelona ethics committee and all participants or, when appropriate, their relatives, gave written informed consent for clinical and post-mortem neuropathological evaluations.

### Statistical Analysis

Descriptive data are reported as median, mean, deviation standard, number or percentage. Duration of RBD was defined as the interval between the reported onset of RBD symptoms (according to patients’ and bed partners’ estimation) and the time of the last follow-up assessment or death. The date of IRBD diagnosis was defined as the date when video-polysomnography demonstrated REM sleep without atonia linked to abnormal behaviors. Follow-up duration was defined as the interval from diagnosis of IRBD to the time of the last visit or death. The onset of a defined neurodegenerative syndrome (PD, DLB, MSA and MCI) was determined as the date when the diagnosis was made according to accepted clinical criteria. Duration of any emerging defined neurodegenerative syndrome was defined as the interval between the time of its diagnosis and the time of the last follow-up assessment or death.

The risk of developing a defined neurodegenerative syndrome in the 174 subjects from the cohort was estimated by means of the Kaplan–Meier method.^14^ Disease-free survival rate was assessed from the date of IRBD diagnosis to the date of the diagnosis of defined neurodegenerative syndrome or to the last follow-up visit for censored observations (subjects who died or were lost to follow-up).

Demographic and clinical variables were compared between the first 44 subjects reported previously [8] and the remaining 130 with Student’s *t* test and χ^2^ test, as appropriate. The risk of developing a neurodegenerative syndrome was calculated for the two groups, and comparisons were assessed with the log rank test.

P values less than 0.05 were considered to be significant. All analyses were done with SPSS version 20.0.

## Results

In our sleep center, 174 individuals were diagnosed with IRBD between November 1991 and July 2013. With the exceptions of the duration of follow-up and the duration of RBD, there were no demographic and clinical differences between the first 44 subjects who were diagnosed between November 1991 and March 2003 and the remaining 130 who were diagnosed between April 2003 and July 2013 (Table 1).

**Table 1:** Comparison between the first 44 subjects who were diagnosed with IRBD between November 1991 and March 2003, and the following 130 who were diagnosed between April 2003 and July 2013.

	Patients diagnosed with IRBDbetween November 1991 andMarch 2003 (n = 44)	Patients diagnosed with IRBDbetween April 2003 andJuly 2013 (n = 130)	P value
Sex (male/female)	39/5	97/33	0.052
Age at estimated RBD onset (years)	62.75±7.34 (45–77)	62.32±7.92 (40–81)	0.744
Age at RBD diagnosis (years)	69.07±6.56 (56–85)	68.57±6.32 (50–85)	0.655
RBD duration (years)	16.50±5.07 (8–31)	10.16±6.65 (1–34)	<0.0001
Follow-up duration after diagnosisof RBD (years)	9.64±3.34 (1–15)	3.50±2.72 (0.1–10)	<0.0001
Self-awareness of abnormal sleep behaviors	28(63.6%)	70(54.3%)	0.279
Unpleasant dream recall	42(95.5%)	118(90.8%)	0.323
Age at diagnosis of emerging definedneurodegenerative syndrome (years)	74.64±5.60 (60–85)	74.31±4.13 (64–80)	0.793
Diagnoses of emerging definedneurodegenerative syndrome (n)	PD = 16 DLB = 15 MSA = 1 MCI = 4	PD = 6 DLB = 14 MSA = 1 MCI = 8	
Estimated risk of conversion after5 years of IRBD diagnosis (%)	34.8	22.6	
Estimated risk of conversion after10 years of IRBD diagnosis (%)	73.4	72.2	

Data are given in number, mean, standard deviation and range. RBD = REM sleep behavior disorder; PD = Parkinson disease; DLB = dementia with Lewy bodies; MSA = multiple system atrophy; MCI = mild cognitive impairment.

Patients were 136 (78.2%) men and 38 (21.8%) women with a median age at estimated RBD onset of 62.0 (range, 40 to 81) years, median age at diagnosis of IRBD of 69.0 (range, 50 to 85) years, and median interval between estimated RBD onset and IRBD diagnosis of 4.0 (range, 0.5 to 30) years. The median age at last visit was 74.0 (range, 57 to 91) years and the median follow-up from IRBD diagnosis to the last visit or to death was 4.0 (range, 0.1 to 15) years.

By the 2013 assessment, 65 (37.4%) patients were diagnosed with a defined neurodegenerative syndrome, 103 (59.2%) remained disease-free, and 6 (3.4%) were lost to follow-up with the diagnosis of IRBD at their last visit. Emerging diagnoses were DLB in 29 subjects, PD in 22 (6 developed dementia after the diagnosis of PD), MCI in 12 and MSA in two. These two subjects were diagnosed with MSA of the cerebellar subtype based on the presence of cerebellar signs plus dysautonomic features (e.g., orthostatic hypotension, urinary incontinence and erectile dysfunction). Follow-up visits disclosed the development of rigid-akinetic parkinsonism unresponsive to levodopa in both, and stridor due to bilateral vocal cord paralysis in one. None of our IRBD patients were diagnosed with pure autonomic failure. In 25 of the 29 subjects who were diagnosed with DLB, a previous period of MCI was recognized, and the median interval between the diagnosis of MCI and DLB was 2.0 (range, 1 to 7) years. Two patients with the diagnosis of MCI developed PD after two years, and they have not developed dementia or visual hallucinations after 2.4 and 3.1 years of follow-up.

**Figure 1:**
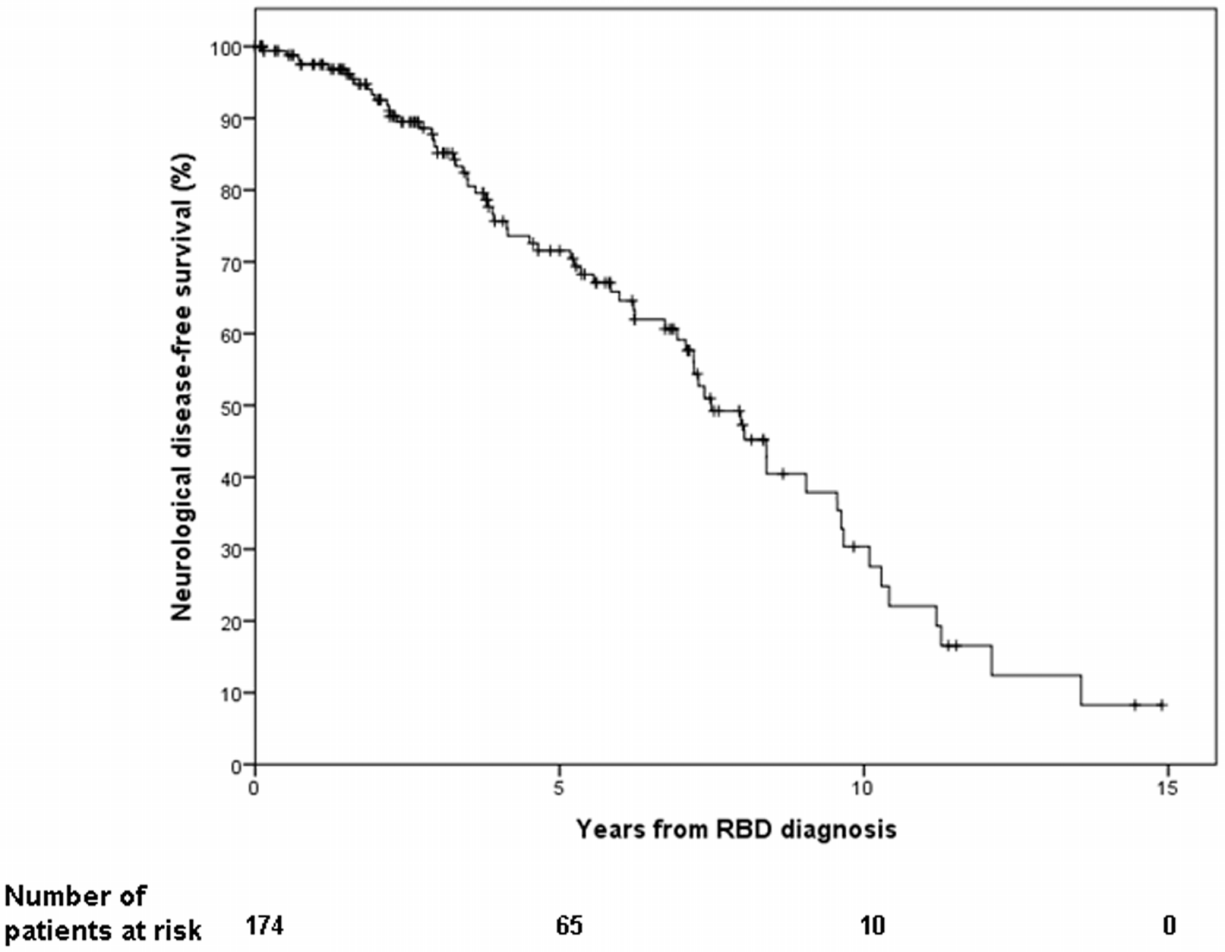
Rates of neurological-disease-free-survival according to the time of IRBD diagnosis in the 174 patients from the cohort.

**Figure 2:**
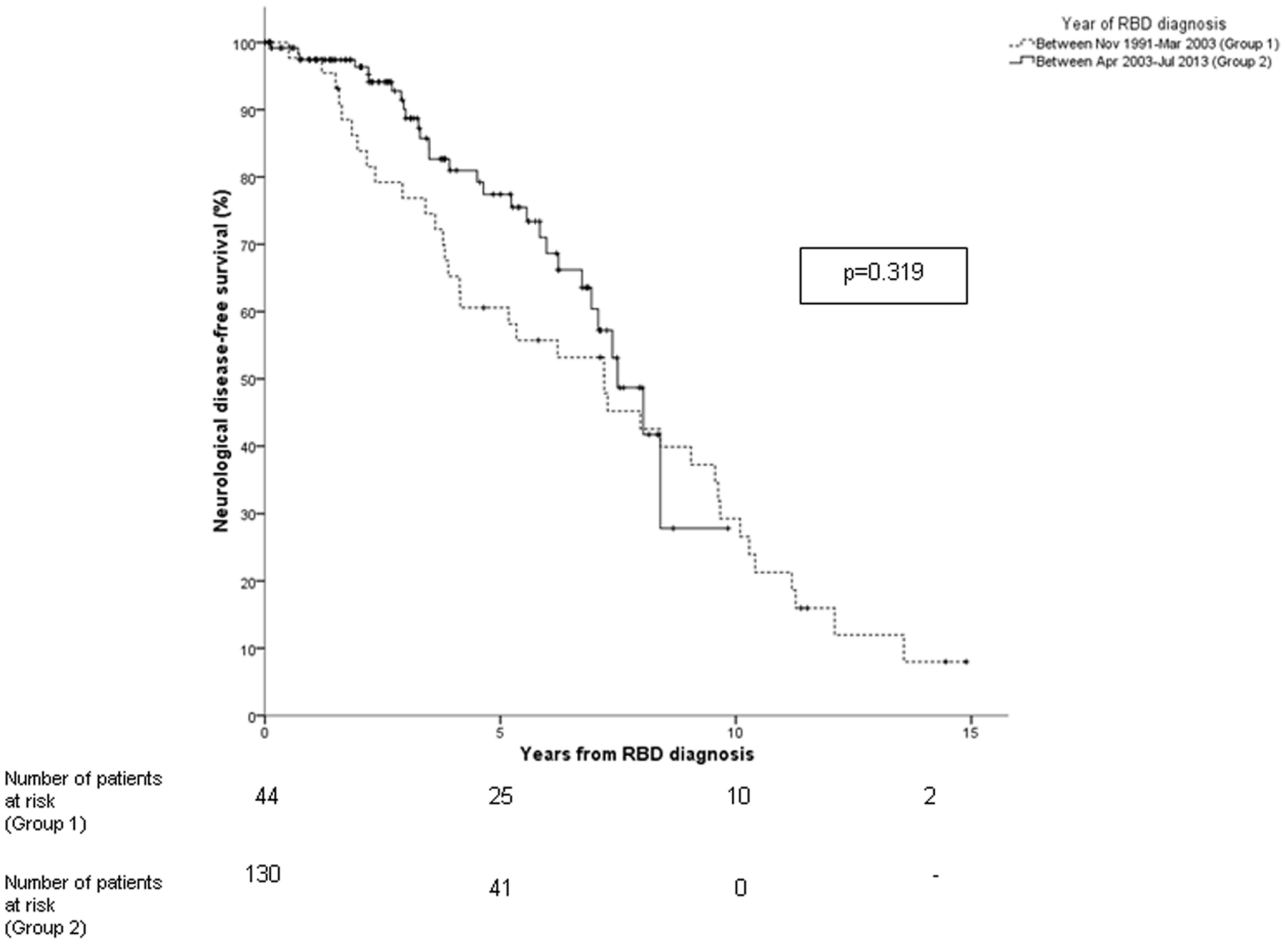
Rates of neurological-disease-free-survival according to the time of IRBD diagnosis in the first 44 patients from the cohort (doted line) and the remaining 130 (continuous line).

In the 65 subjects diagnosed with a defined neurodegenerative syndrome, the median interval between estimated RBD onset and diagnosis of a defined neurodegenerative syndrome was 11.0 (range, 2 to 24) years, and the median interval between IRBD diagnosis and diagnosis of a defined neurodegenerative syndrome was 4.0 (range, 0.5 to 13) years. The risk of a defined neurodegenerative syndrome from IRBD diagnosis was 33.1% at five years, 75.7% at ten years, and 90.9% at 14 years (Figure 1). The median conversion time in our sample was 7.5±0.5 years (95% CI 6.5 to 8.4). No difference in the risk of developing a neurodegenerative syndrome was found between the first 44 patients and the subsequent 130 patients (p = 0.319) (Figure 2).

During the follow-up period, 32 (18.4%) subjects were deceased. Twenty-four of them were clinically diagnosed during life with a defined neurodegenerative syndrome (11 with DLB, 10 with PD and 3 with MCI), and the remaining eight had the diagnosis of IRBD at their last follow-up visit. Six patients with the antemortem diagnoses of DLB (n = 3), PD (n = 2, one of them with associated dementia) and MCI (n = 1) were brain donors. In two of them (the one with PD without dementia, and the one with MCI) the peripheral autonomic nervous system was also available for examination. In the brain, all six cases presented variable neuronal loss, gliosis and alpha-synuclein immunoreactive Lewy pathology (Lewy bodies and Lewy neurites) in vulnerable regions such as the olfactory bulb, dorsal motor nucleus vagal nerve, central raphe nucleus, the nuclei that regulate REM sleep muscle atonia (gigantocellular reticular nucleus, pedunculopontine nucleus and coeruleus/subcoeruleus complex), substantia nigra pars compacta, limbic structures (amygdala and anterior cingulate cortex), and nucleus basalis of Meynert. Lewy pathology was also detected in the neocortex of the three patients with the antemortem diagnoses of DLB and of the PD patient who developed dementia, corresponding in all of them to Lewy body Braak stage 5 [15]. In the patient with the antemortem diagnosis of PD who did not develop dementia and in the patient with MCI, Lewy pathology was observed in the olfactory bulb, entire brainstem and amygdala, preserving neocortical areas (Lewy body Braak stage 4) [15]. In these two patients, α-synuclein aggregates were observed in several regions of the peripheral autonomic nervous system such as the paravertebral sympathetic chain, epicardial fat tissue ganglia and myenteric plexus. As commonly seen in patients with Lewy body disorders [16], [17], concomitant pathologies were observed and included 1) Alzheimer type pathology in two of the donors with the clinical diagnosis of DLB, 2) small vessel disease with microvascular lesions in two DLB cases and the PD subject without dementia, and 3) argyrophilic grain pathology in the PD patient with dementia, in one DLB case and in the MCI case.

## Discussion

This is the largest study with long term follow-up of IRBD. In a cohort of 174 patients that were closely followed during a median period of 4.0 years, survival curves showed that the estimated risk of a defined neurodegenerative syndrome from the time of IRBD diagnosis was 33.1% at five years, 75.7% at ten years, and 90.9% at 14 years. Patients were clinically diagnosed with PD, DLB, MSA and MCI. In patients who underwent postmortem brain examination, the antemortem diagnoses of PD and DLB were confirmed showing neuronal loss and widespread Lewy-type pathology in selective brain regions. The patient with MCI and lack of parkinsonism and dementia also had widespread Lewy type pathology. The current study confirms our first observation [8] that the majority of patients with IRBD that seek medical attention are eventually diagnosed with a synucleinopathy. Ours is a homogenous cohort where demographics, clinic characteristics and survival curves are similar between the 44 subjects diagnosed between 1991 and March 2003 [8] and the subsequent 130 subjects diagnosed between April 2003 and July 2013.

Similar results have been found by two other studies where IRBD patients diagnosed in sleep tertiary centers were clinically followed. The nature of disorders (synucleinopathies) evolving from these two cohorts is very similar to what we have found in our study. In the first study, Schenck et al. found that parkinsonism developed in 11 of 29 (38%) IRBD subjects nearly four years after the diagnosis of IRBD and almost 13 years after the onset of RBD [4]. After 16 additional years of follow-up, 21 (72.4%) IRBD patients from the original cohort where diagnosed with PD in 13 cases, DLB in three, MSA in two, unspecified dementia in one, and Alzheimer’s disease with autopsy-confirmed combined Alzheimer’s disease pathology plus Lewy pathology in two. Three IRBD subjects were lost during the follow-up period [5]. In this study, the presence of MCI was not evaluated and the risk of disease was not examined using survival curves and statistical analysis. In a second series, Postuma et al. found that 26 of 93 (28%) IRBD patients developed a neurodegenerative disorder after a mean follow-up of five years; PD in 14, DLB in seven, dementia that met clinical criteria for Alzheimer’s disease (but also for possible DLB) in four, and MSA in one. Survival curves showed that the estimated risk of a neurodegenerative disease after RBD diagnosis was 17.7% at five years, 40.6% at 10 years, and 52.4% at 12 years [6]. When compared with our study, the lower conversion rate and the lower estimated risk of developing neurodegenerative disease found by Postuma et al. may be explained by methodological aspects. In the study by Postuma et al. the design was retrospective (while ours was prospective), the status of 15 patients was assessed through telephone interviews (while all of our patients were assessed by in-person examinations even examining them at their homes and nursing-homes), 20 (17.7%) of their initial 113 patients where lost during the follow-up (while we lost only six −3.4%− of 174 subjects), neuropathologic confirmation of a disease was not provided (in our study this was done in six subjects), the mean age at the time of the study was 65 years (while in ours it was 74 years), and MCI was not considered as a disease outcome.

Our findings indicate that there is a strong and specific association of IRBD with PD and DLB, and that this parasomnia can be considered in most cases a prodromal manifestation. In PD, other clinical features known to precede the motor symptoms such as hyposmia, constipation, depression and hypersomnia are much less specific since only a small number of individuals with these symptoms are eventually diagnosed with PD [18], [19]. This positions IRBD as an optimal target population to study the prodromal stage of the synucleinopathies and eventually to test disease-modifying strategies in these disorders before motor and cognitive changes have developed.

The findings of our study have clinical relevance and research implications. The investigation of patients with IRBD offers a unique opportunity to obtain information of the aetiology, pathogenesis and progression of the prodromal stage of the synucleinopathies. In IRBD, clinical, neuroimaging, biochemical, proteomic and genetic studies will help to better understand the mechanisms of neurodegeneration and to design interventions with potential disease modifying properties. To date, several studies have added weight to the concept that IRBD represents in most cases an evolving synucleinopathy in its earliest phases. Several studies have found that IRBD patients display clinical (e.g., hyposmia [20], depression [21], autonomic dysfunction [21]) and neuroimaging (e.g., decreased dopamine transporter binding in the striatum [22], substantia nigra hypechogenicity [22], increased mean diffusivity in the pons [23]) abnormalities which are characteristic features of the synucleinopathies. Overall, these studies suggest that at the time IRBD is diagnosed a neurodegenerative process is already widespread in the nervous system. Of these abnormalities, the combination of hyposmia with colour vision impairment [20], and the combination of substantia nigra hyperechogenicity with decreased striatal dopamine transporter uptake [21] may identify those IRBD patients who are at increased short-term risk for being diagnosed with a synucleinopathy according to accepted clinical criteria. Prospective studies have further shown that while colour vision and olfactory dysfunction [20], [24] remains stable over time, dopamine transporter imaging shows progressive decline in striatal tracer uptake [25]. Thus, dopamine transporter imaging, and not olfactory and colour vision functions, serve better as a marker of progression in IRBD.

The finding that IRBD patients are later frequently diagnosed with DLB and PD with associated dementia suggests that both disorders may represent different phenotypes from the same condition. MCI is an intermediate stage between normal cognitive function and dementia where individuals have cognitive complaints, deficits in neuropsychological tests and their daily living activities are preserved [13]. In our study MCI was considered a disease outcome. This is because in the particular setting of IRBD, MCI can be considered an abnormal condition which frequently evolves into DLB and PD. This is based on the following data. First, MCI occurs in nearly 20% of untreated PD patients at the time of initial diagnosis [26], and predicts conversion to dementia [27]. Of note, two our MCI patients have developed parkinsonism but not dementia or hallucinations to date. Second, most IRBD patients seen in sleep centers who develop MCI are eventually diagnosed with DLB [8]. Third, IRBD patients that develop MCI but no dementia show markers of a synucleinopathy such as decreased striatal dopamine transporter, hyperechogenicity of the substantia nigra and hyposmia [8]. Fourth, in patients with MCI with comorbid RBD who later develop dementia, postmortem examination shows Lewy-body pathology [28]. Finally, in a population-based study involving 651 individuals, RBD conferred a 2.2-fold increased risk of developing MCI over a 4-year period of observation [29]. The above data is supported by our current observation in one patient who developed MCI (and not dementia or parkinsonism) and neuropathology demonstrated neuronal loss and widespread alpha-synuclein related Lewy body pathology involving the peripheral nervous system, olfactory bulb, entire brainstem (including the substantia nigra) and the limbic system.

Limitations of our study should be acknowledged. Our study did not include a control group of healthy subjects without RBD who were followed-up on during a similar time period. In the present study, the proportion of IRBD subjects that developed a neurological condition was 37% over a median follow-up period of 4 years. This proportion is greatly superior to the estimated prevalence of these neurological disorders in a population of individuals of similar age and sex distribution [30], [31], [32], [33]. Our cohort comprises patients who were self-referred to a tertiary sleep center. It is uncertain if our observations can be generalized to the individuals with IRBD in the general population that do not seek medical attention. Strenghts of our study include confirmation of RBD by polysomnography in all cases, a large number of study subjects, and a long and close follow-up (which included home and home-nursing visits when needed) reflected by a low number (3%) of patients who were lost. In addition, pathological confirmation was available in six cases and clinical outcome (disease free or diagnosed with a defined neurodegenerative syndrome) was in all cases determined by in-person assessment through clinical history and neurological evaluation.

In summary, our study shows that a majority of IRBD patients with prolonged follow-up are diagnosed with the synucleinopathies PD, DLB and MSA. IRBD represents the prodromal period of these conditions. Our findings in IRBD have important implications in clinical practice, in the investigation of the early pathological events occurring in the synucleinopathies, and for the design of future trials with potential disease-modifying agents.
